# Anesthetic management of patients undergoing bariatric surgery: two year experience in a single institution in Switzerland

**DOI:** 10.1186/1471-2253-14-125

**Published:** 2014-12-18

**Authors:** Bastian Lindauer, Marc P Steurer, Markus K Müller, Alexander Dullenkopf

**Affiliations:** Department of Anesthesia and Intensive Care, Kantonsspital Frauenfeld, Pfaffenholzstrasse, 48501 Frauenfeld, Switzerland; Department of Anesthesia and Perioperative Care, University of California, San Francisco, USA; Department of Surgery, Kantonsspital Frauenfeld, Frauenfeld, Switzerland

**Keywords:** Anesthesia, Complications, Bariatric surgery, Obesity

## Abstract

**Background:**

In the field of anesthesia for bariatric surgery, a wide variety of recommendations exist, but a general consensus on the perioperative management of such patients is missing. We outline the perioperative experiences that we gained in the first two years after introducing a bariatric program.

**Methods:**

The perioperative approach was established together with all relevant disciplines. Pertinent topics for the anesthesiologists were; successful airway management, indications for more invasive monitoring, and the planning of the postoperative period and deposition. This retrospective analysis was approved by the local ethics committee. Data are mean [SD].

**Results:**

182 bariatric surgical procedures were performed (147 gastric bypass procedures (GBP; 146 (99.3%) performed laparascopically). GBP patients were 43 [10] years old, 78% female, BMI 45 [7] kg/m^2^, 73% ASA physical status of 2. 42 patients (28.6%) presented with obstructive sleep apnea syndrome. 117 GBP (79.6%) patients were intubated conventionally by direct laryngoscopy (one converted to fiber-optic intubation, one aspiration of gastric contents). 32 patients (21.8%) required an arterial line, 10 patients (6.8%) a central venous line. Induction lasted 25 [16] min, the procedure itself 138 [42] min. No blood products were required. Two patients (1.4%) presented with hypothermia (<35°C) at the end of their case. The emergence period lasted 17 [9] min. Postoperatively, 32 patients (21.8%) were transferred to the ICU (one ventilated). The other patients spent 4.1 [0.7] h in the post anesthesia care unit. 15 patients (10.2%) required take backs for surgical revision (two laparotomies).

**Conclusions:**

The physiology and anatomy of bariatric patients demand a tailored approach from both the anesthesiologist and the perioperative team. The interaction of a multi-disciplinary team is key to achieving good outcomes and a low rate of complications.

**Trial registration:**

DRKS00005437 (date of registration 16^th^ December 2013)

## Background

Obesity represents a significant and growing problem around the globe [1]. Aside from the impairment of an individual patient, the negative consequences impose a significant economic burden for many health care systems [2].

For over a decade, bariatric surgical procedures have established themselves as a way to achieve a permanent weight reduction for a large number of patients [3].

This patient population represents a particular challenge for the anesthetist, with multiple publications offering a wide range of recommendations on the matter [4–8]. Additionally, there are also large differences in terms of the data that outline the perioperative process times [9–12]. All of this results in difficulty to establish such a program and predict its trajectory at the early stages.

We report the perioperative experience two years after the start of such a bariatric program at our single Swiss institution.

## Methods

In early 2011 a multidisciplinary obesity program was launched at the Kantonsspital Frauenfeld (KSF) (General Hospital Level 2, 270 beds, about 8400 anesthetics per year). The program encompassed bariatric surgical care, psychiatric/psychosomatic patient guidance, nutrition counselling, gastro-enterological and cardiac work-up, as well as the follow-up and physical therapy.

Anesthesia and intensive care medicine physicians were involved early in the planning process. In collaboration with the surgical team, a perioperative approach for patient care was developed for each individual patient.

From an anesthesia perspective, the key points of this concept were the characterization of preoperative testing including laboratory tests. A detailed cardiac evaluation was obtained for all patients that were either over 55 years, had a BMI >50 kg/m^2^, an exercise tolerance of <4 MET or any significant cardiac history. An evaluation by a pulmonologist including lung function testing and screening for obstructive sleep apnoea (OSA) were performed for all patients, if not previously diagnosed. The anesthetist in the preoperative clinic saw the patients about two weeks preoperatively or on the day before surgery. The main focus was on the detection of any form of OSA and its pre-existing treatment. All patients were informed and consented about the possibilies of an awake fiber-optic intubation, an arterial line (AL), a central venous catheter (CVC) and a postoperative stay in the intensive care unit (ICU).

The nil per os (NPO) time for all patients was six hours fasting for solid food, and two hours for clear liquids. Midazolam 7.5 mg po was given preoperatively, unless the patient had a BMI >40 kg/m^2^, or was diagnosed with OSA, in order to minimize the risk of perioperative hypoventilation. Patients were instructed to take their baseline medication with the exception of ACE inhibitors or AT-II antagonists. In patients with a history of gastro-esophageal reflux, a therapy with proton pump inhibitors (PPI) was initiated if not already in place.

For the anesthetic induction, peripheral venous access was established. When non-invasive blood pressure measurements were reliably feasible, no AL was placed. The choice of the individual airway management technique (conventional direct laryngoscopy vs fiber-optic intubation either awake, as a rapid sequence induction or asleep) was at the discretion of the assigned anesthesia attending. All conventional intubations were performed in Anti-Trendelenburg position in order to reduce the risk of regurgitation and aspiration of gastric contents. Preoxygenation was performed with the patient breathing spontaneously via face mask (FiO_2_ = 1.0) until the end-expiratory FiO_2_ was at least 0.8. Propofol was used for induction, followed by BIS monitoring guided Propofol infusion and/ or Sevoflurane for anesthesia maintenance, supplemented with Fentanyl and Remifentanil. Propofol was dosed as target controlled infusion (TCI) with a range of 2.2 to 4 mcg/ml and was supplemented by Sevoflurane, as needed. Sevoflurane was used when the Propofol infusion resulted in hypotension and had to be reduced to below 2.2 mcg/ml or when despite a Propofol TCI of 4 mcg/ml the anesthetic depth was insufficient, as indicated by the BIS monitoring.

Cefazolin, 1.5 g IV served as the antibiotic prophylaxis. Paralysis for intubation was achieved with either Succinylcholine or Atracurium,. Atracurium was the paralytic of choice for subsequent paralysis. After intubation a second large bore peripheral IV catheter (≥17G) was placed. In cases where peripheral vascular access was difficult, a CVC was inserted. For the surgical procedure, the patient was positioned in a modified, semi-standing beach chair position. In order to prevent deep venous thrombosis, sequential compression devices were applied intraoperatively, followed by low molecular weight heparin sc on the first postoperative evening as prescribed by the surgeon, in combination with early mobilization of the patients. An inspiratory oxygen fraction (FiO_2_) of 0.6 and a positive end expiratory pressure (PEEP) of 5 cm H_2_O were the standard initial ventilator settings, while using either pressure controlled or volume controlled ventilation (target for P_max_ ≤30 cm H_2_O, and tidal volume 6 ml/kg). Paralysis at the end of the procedure was checked with quantitative TOF-determination (TOF-Watch; Organon, Dublin, Ireland). If the TOF-ratio was less than 0.9, Glycopyrrolate combined with Neostigmine was used to reverse the neuromuscular blockade. Postoperative nausea and vomiting (PONV) was pre-emptively treated according to an escalating protocol based on the Apfel risk score [13]. In order to be extubated after the procedure, a patient’s body temperature had to be 35.0°C or higher. The emergence and extubation were performed in a separate dedicated room. The multimodal postoperative pain regime was initiated 20 min before end of surgery with a combination of 5 mg of morphine iv, paracetamol IV 4 × 1 g/d, metamizole IV 5 g/24 h, controlled-release oxycodone and naloxone (Targin®) po 2 × 10/5 mg, and morphine IV 2 – 5 mg as rescue medication. Non-steroidal anti-inflammatory drugs were omitted, and patients were kept NPO for the first postoperative night, except for the mentioned Targin®.

Elective patients with an uneventful surgical and anesthetic course were recovered in the post anesthesia care unit (PACU). Patients with OSA and no continuous positive airway pressure (CPAP) therapy were admitted to the ICU postoperatively to provide continuous monitoring for the first post-operative night. Pre-existing CPAP therapy was continued after extubation, using the patient’s usual setup and adjusting the settings as clinically needed in the PACU.

The institutional ethics committee of the Canton of Thurgau, Switzerland, approved the retrospective analysis of the anesthesia documents and medical records of all patients undergoing bariatric surgery in the first two years after the establishment of the new bariatric program at the Kantonsspital Frauenfeld (KEKTG/PEB2013/09). The study was registered with the German registry for clinical studies (http://www.drks.de; DRKS00005437). Essential anesthesia and perioperative data and complications were collected and investigated.

The surgical procedures of the patients in the bariatric program were quite heterogeneous. Therefore we mainly investigated the data of patients undergoing a gastric bypass procedure (GBP). We did not include any follow-up interventions of cosmetic nature.

The data are presented descriptively as mean [standard deviation].

## Results

During the two year observation period (2011–2013), after initiating the bariatric program, a total of 182 bariatric operations were performed, 178 of them laparascopically.

Patients had a mean age of 44 [10] years, were predominantly female (79.7%), with a BMI of 43 [8] kg/m^2^. The surgical procedures were quite heterogeneous and had operation times of 30 min for a laparoscopic removal of a gastric band up to 425 min for a laparotomy for a complex revision case (mean 130 [51] minutes).

Because of that heterogeneity, we decided to only include patients that underwent gastric bypass procedures (GBP) in the further analysis. The total number of GBP was 147, all but one of them were performed laparascopically. 19 cases (12.9%) needed an additional procedure at the same time (18 removal of a gastric band, 1 cholecystectomy).

Table 1 shows the demographic data of the GBP patients.

**Table 1:** **Demographic data and comorbidities of patients undergoing gastric bypass surgery**

Sex	Female – Male; n	115 – 32
Age	Years	43 [10]
Height	m	1.67 [0.09]
Weight	kg	125 [23]
BMI	kg / m ^2^	45 [7]
Patients presenting with		
Arterial hypertension	n (%)	67 (45.6)
OSA	n (%)	42 (28.6)
Diabetes mellitus	n (%)	33 (22.4)
GERD	n (%)	81 (55.1)
Smoking habit	n (%)	68 (46.3)

Data are number, n (% of patients); or mean [standard deviation].

OSA = obstructive sleep apnea; GERD = gastro-esophageal reflux disease.

All patients underwent thorough preoperative evaluations. 107 patients (72.8%) were classified with an ASA physical status of 2, 39 (26.5%) with ASA 3 and 1 patient (0.7%) with ASA 4. 67 patients (45.6%) presented with arterial hypertension, 42 patients (28.6%) with OSA (29 (69.0%) of them with CPAP therapy), 33 patients (22.4%) had (mostly non-insulin dependent) diabetes mellitus, 81 (55.1%) had gastro-esophageal reflux disease, 68 patients (46.3%) were smokers, with a mean of 24 [20] pack years. 92 patients (62.6%) reported no regular medication.

In 101 patients a chest X-ray was performed as part of their preoperative work up. This resulted in 13 patients (12.8% of studies) demonstrating pathologic findings, mostly in the form of either atelectasis or left ventricular enlargement. In 143 patients a preoperative ECG was obtained, it showed abnormal findings in 26 patients (18.2%), 4 of them had a prolonged QTc interval. Forty-nine patients received a preoperative echocardiography; this resulted in 21 patients (42.9%) with a pathological result. Two patients presented with a moderate valvulopathy, one patient had a severely impaired LVEF of 25%. A stress test was performed in 96 patients, and resulted in 24 patients (25%) with abnormalities, 2 patients had clear signs of ischemia during exercise. Preoperative gastroscopy (137 patients) was pathological in 91 patients (66.4%) (e.g. axial hiatal hernia, GERD). The other preoperative investigations revealed abnormal findings in 74 patients (50.3%), the majority of them were found during the abdominal ultrasound. The majority of patients (142, 96.6% of gastric bypass patients) were admitted to the hospital at least one day prior the surgery.

In 115 patients (78.2%) the non-invasive blood pressure measurement was considered sufficient, in 32 patients (21.8%) an AL was placed for blood pressure measurement. A second large bore peripheral IV was placed in 137 patients (93.2%), 10 patients (6.8%) received a central venous catheter (CVC).

Twenty-nine patients (19.7%) were intubated fiber-optically, 26 of them awake, 3 patients after induction of general anesthesia. One of them was a difficult intubation (required 3 attempts). 117 patients (79.6%) were intubated with direct laryngoscopy, 103 of them with a rapid sequence induction. In 87.4% of the conventional intubation cases, patients presented with a Cormack-Lehane grade 1, 8.1% had a grade 2, 4.5% a grade 3 and in one case (0.9%) the airway had to be secured unplanned with the fiber-optic. One patient (0.7%) experienced an episode of aspiration of gastric contents that remained without consequences.

In 146 patients (99.3%) anesthesia was induced with Propofol and 145 patients (98.6%) received a Propofol infusion (2 [0.7] g) as main anesthetic for the case. Pain management was achieved with fentanyl (0.6 [0.2] mg) and remifentanil (2.0 [0.4] mg). 26 of the patients (17.9%) also received a supplementation of their anesthetic with Sevoflurane. In 2 patients (1.4%) the anesthesia was maintained with Sevoflurane only. Fluid homeostasis was achieved with 1700 [600] ml of Ringerfundin (B. Braun Medical, Sempach, Switzerland), and 400 [300] ml of HES 130/04 (Voluven; Fresenius Kabi, Oberdorf, Switzerland). In 59 cases (40.1%) the patient’s blood pressure had to be supported pharmacologically, mainly due to the beach chair positioning of the patients. 52 times boluses of Ephedrine were used and in 7 cases Norepinephrine was given in boluses. One patient (0.7%) received Atropine to counteract a relevant bradycardia. In 24 cases (16.3%) it was necessary to medically address hypertension. This was done using Clonidine in 21 instances, and for 3 patients Urapidil was the medication of choice.

In 63 patients (42.9%) an intra-operative PEEP in excess of 5 cm H_2_O was required (maximum 11 cm H_2_O).

The perioperative process times are shown in Table 2.

**Table 2:** **Perioperative process times of gastric bypass surgery**

Anesthesia induction time	Min	25 [16]
Surgery time	Min	138 [42]
Anesthesia emergence time	Min	17 [9]
Total anesthesia time	Min	238 [47]
Patients requiring re-intubation	n	0
Patients requiring blood products intraoperatively	n	0
PACU stay	h	4.1 [0.7]
ICU stay	h	17 [12]
Patients requiring blood products during hospital stay	n	4 (2.7%)
Patients requiring revision surgery	n	15 (10.2%)
Hospital stay	Days	6 [2]

Data are number, n (% of patients); or mean [standard deviation].

PACU = post anesthesia care unit, ICU = intensive care unit.

No blood products had to be administered for any case. Four patients (2.7%) required a blood transfusion in the subsequent course of their hospital stay.

At the end of procedure 116 patients (78.9%) needed reversal of their paralysis. The core body temperature was 36 [0.5] °C. Two patients (1.4%) could not be maintained above 35°C during the procedure, one of these patients was transferred to the intensive care where he was extubated one hour later. All extubations were unproblematic, no patient had to be re-intubated.

Postoperatively 115 patients (78.2%) were transferred to the post anesthesia care unit to recover from the procedure, 32 patients (21.8%) were transferred to the ICU. 15 of those admissions (10.2% of all gastric bypass patients) were planned preoperatively due to untreated OSA. None of these patients needed anything other than supplemental application of nasal oxygen. The reasons for unplanned ICU admission was hemodynamic instability in 6 patients, insufficient oxygenation in one patient, hypothermia in another patient (<35°C). The average length of stay in the post anesthesia care unit was 4.1 [0.7] hours, patients that went to the ICU remained there for 17 [12] hours.

During the observation period 15 (10.2%) of the gastric bypass patients required additional surgery (4 major operations), two (1.4%) of them were laparotomies. Eight patients (5.4%) needed surgical revision of some form of an incisional or internal hernia. Three patients (2.0%) presented with some form of postoperative hemorrhage that required the transfusion of blood products, 2 patients (1.4%) developed pneumonia, and one of them required non-invasive ventilation. There was zero in-hospital mortality. The average length of hospital stay was 6 [2] days.

## Discussion

This publication reflects on the perioperative and anesthetic experience two years after starting a bariatric program. Retrospectively, the patient’s medical charts and anesthesia records were analyzed with emphasis on the bariatric surgery and any follow up procedures. All the essential process times and metrics were collected to create insight into the effects of implementing a surgical bariatric program from an anesthesia standpoint.

Our main finding was that the bariatric patient population presents with its unique challenges. When managed appropriately in an interdisciplinary fashion, anesthesia can be delivered safely to this patient population. The preoperative evaluation of these patients should be conducted based on an individual’s need rather than relying on standardized test batteries.

Anesthesia for morbidly obese patients is generally regarded as being associated with increased risks [8, 14]. Accordingly, there are various contributions on the anesthetic and perioperative management in this patient group [4–7, 15]. They include the preoperative assessment, especially with regards to the airway management and monitoring strategies, the intra-operative phase, the emergence from anesthesia and the postoperative phase. Since bariatric surgery is being used in a more widespread manner, caring for morbidly obese patients is becoming more common for many institutions [3]. The surgical procedures are well illustrated by Ogunnaike et al. [6], e.g.

### General

When dealing with the anesthetic considerations for bariatric surgery, it quickly becomes clear that it takes more than good surgeons and anesthetists to have a successful program. The patients need involvement of additional specialists such as Internal Medicine, Psychiatry, Physical Therapy etc. [3]. In addition, emphasis has to be put on the follow up care since a significant proportion of the patients will require additional surgical interventions. This affected 10% of our patients after gastric bypass surgery. Not included in this number were cosmetic follow-up operations such as abdominoplasty after the weight loss. Possible complications such as anastomotic leakage or internal hernia that require urgent care have to be considered when setting up the infrastructure.

### Preoperative evaluation

Ideally, the extent of the preoperative diagnostics are tailored for each patient [7, 10, 15–17]. Heinrich et al. [10] reported, that approximately 20% of their patients received preoperative pulmonary function tests, which in return showed pathological values in over 90% of the cases. Such a streamlined approach is not always feasible, resulting in more generous recommendations for preoperative testing [4, 6]. Overall, the reported incidences of comorbidities such as hypertension or diabetes mellitus are lower than maybe expected [9, 12]. Great emphasis is generally put on the preoperative detection of an obstructive sleep apnoea syndrome, which mainly results in consequences for the postoperative phase, but also can influence the course before the surgery [7]. There seems to be a general consensus on the evaluation of the intubation anatomy, but no uniform approach exists [7, 18].

In our cohort, the vast majority of patients presented with an ASA physical status of 2. Arterial hypertension, diabetes mellitus and other overweight associated comorbidities were relatively often encountered. At the same time they barely ever influenced the perioperative approach. In collaboration with the Cardiology department of our hospital we have defined a pathway for the subset of patients that need to be seen by a cardiologist preoperatively. As a result we had only 40% of the performed echocardiograms and 25% of the stress tests with pathological findings. These results were instrumental to evaluate the general risk, determine the need for intraoperative monitoring, and to clarify the necessity of postoperative observation in the intensive care unit. No preoperative test results were so severe or unexpected that the surgical procedure had to be cancelled. Preoperative examinations such as gastroscopy or abdominal ultrasonography were used to plan the surgical procedure and did not affect the anesthetic approach.

### Anesthetic

A key factor for the perioperative course of those patients was the duration of anesthesia, which in return consists mostly of the duration of surgery and the expertise of the surgeon [19]. Various authors report very different times for gastric bypass surgery; a median of 241 min for Leykin [12], 160 min for Heinrich et al. [10], 120 min in a review by Shang and Beck [15], and a quick 40 min for Jacobsen et al., and Bergland et al. in a dedicated center in Oslo, Norway [9, 11]. The group around Jacobsen [11] additionally showed that the duration of the emergence from anesthesia could be substantially reduced as a result from standardization of the surgical procedure that also produced reliable waypoints for the anesthetist. In our cohort, we note a wide distribution of the times for anesthesia induction and emergence. This fact should be given additional consideration when coordinating the operating room resources.

The intraoperative monitoring of patients undergoing GBP is very heterogeneous. In many institutions the insertion of an arterial cannula for blood pressure measurement is standard [5] or at least generously applied [7, 10, 15]. A similar approach to the liberal usage of central venous access seems also typical [10]. At the same time there are also data from large bariatric centers in which invasive monitoring is largely avoided [9]. In our series, the non-invasive blood pressure measurement was clinically judged to be reliable in 80% of patients. Also, in 94% of the cases, 2 adequate peripheral venous catheters were successfully inserted.

During induction of anesthesia the main focus was on the airway management. The importance of proper patient positioning and pre-oxygenation is well described [7]. Depending on the training background, the fiber-optic intubation in the awake patient has a high significance [4, 12]. When patients are intubated with conventional, direct laryngoscopy, they are reported to either have roughly the same [9, 18, 20] or slightly more difficult intubation condition than comparable cohorts [5, 10]. In our institution, the details of the airway management were at the discretion of the attending anesthesiologist. In one of our reference hospitals (Kantonsspital St. Gallen, Switzerland, http://www.kssg.ch), the fiber-optic intubation in the awake patient is strongly propagated for patients with a BMI >35 kg/m^2^ which partially explains the rather large share of almost 20% of our patients being intubated in such fashion. The conventional laryngoscopies resulted in 80% in complete visibility of the glottis (Cormack-Lehane grade I). In one patient the conventional intubation failed, highlighting the need to define an appropriate contingency plan. In all cases with conventional laryngoscopy we performed mask ventilation after the induction of anesthesia. In our rapid sequence protocol we set the inspiratory pressure to a maximum of 15 cm H_2_O and ventilated the patient’s lungs about 4 times per minute.

In most publications the anesthesia maintenance is described as being accomplished with volatile anesthetics or balanced anesthesia [10, 11], but Propofol-based anesthesia regimes are also mentioned [7, 12]. In our patients we almost exclusively used Propofol, which provided adequate hemodynamic stability and a rapid and uncomplicated emergence. Regarding our emergence times, it should be noted that in our setting the extubation took place in a dedicated area in the OR for the majority of cases. The rate of intraoperative complications is described to be around 5%, of those roughly a fifth can be regarded as anesthesia-related [14]. During the procedure the adequate oxygenation of the patient is of main concern [7]. Recruitment maneuvers and increased PEEP levels were described as effective, but are not of great sustainability [21]. Accordingly, it was deemed necessary to increase the PEEP level above the default setting of 5 cm H_2_O in 40% of our patients.

No blood products had to be administered intraoperatively in any case. Due to the relative difficulty to access the patients during the procedure we performed a type and screen on all of them, so we would have blood products readily available should the need arise. Because of the long duration of the procedure we put a clear emphasis on the temperature management and consequently only had two of our patients (1.4%) that had to be extubated delayed due to hypothermia. The emergence from anesthesia was generally unproblematic, mostly due to the appropriate planning and consistent avoidance of residual paralysis.

### Postoperative care

The rate of postoperative ventilation requirement and the surveillance in either a 24-hour post anesthesia care unit or an intensive care unit varies considerably. Nishiyama et al. [5] for example report over 25% patients requiring postoperative ventilation in their case series, Leykin [12] had a rate of about 4%. Heinrich et al. [10] monitored over 50% of their gastric bypass patients for more than 24 hours in a dedicated intermediate care unit and had over 10% of the patients intubated during that period. On the other hand, Jacobsen et al. [11] reported that only 3 out of 2,000 patients required surveillance in the intensive care unit over a period of 5 years, whereas most patients only spent 2–4 h in the post anesthesia care unit. In our setting, which is devoid of a step down unit, a patient with untreated sleep apnoea syndrome was considered to be an indication for postoperative admission to the ICU, in accordance to the ASA guidelines for postoperative monitoring in OSA patients. This explains half (10% of all patients) of the ICU admissions. However, no interventions regarding to the OSA were necessary. The other 10% of the patients had varying reasons for their stay in the ICU.

Pain management can be particularly challenging in bariatric surgery patients. In our retrospective analysis it was not possible to evaluate standardized data for mobilization of patients or pain assessments. However, it should be mentioned that in no patient an intervention of our peri-operative pain service (e.g. regional anesthesia or iv patient controlled analgesia) was necessary.

In general, the anesthetic literature on bariatric surgery appears to be very heterogeneous. This is reflected in the volume of reported operations performed in each center, the characteristic process times and also in the anesthetic management. However, some recommendations found in the literature seem rather laborious and intricate.

The mid-term goals for our perioperative approach are aiming towards a more formalized fast-track bariatric surgery regime. This should be facilitated by a reduction in the surgical times and enhanced interdisciplinary collaboration with focus on the essentials [9, 11]. Additionally, the risk stratification of airway difficulties should be more standardized and the extent of preoperative testing re-considered. Providing muscle relaxation with rocuronium together with its specific reversal with sugammadex certainly has a potential place in this context [22]. The outcomes of the proposed strategies with respect to prophylaxis of PONV and pain management have to be followed in more detail. Another interesting detail to be investigated in the future is the possible difference in perioperative risk in patients being “just obese” compared to those being diagnosed as having metabolic syndrome [23, 24].

## Conclusions

Bariatric anesthesia comes with its unique challenges, but when approached in an thoughtful and interdisciplinary fashion it becomes safely manageable. In addition, the need for some urgent follow-up operations and appropriate postoperative monitoring capabilities have to be accounted for at all times.

## References

[1] Bibiloni MD, Pons A, Tur JA (2013). Prevalence of Overweight and Obesity in Adolescents: A Systematic Review. ISRN obes.

[2] Apovian CM (2013). The clinical and economic consequences of obesity. Am J Manag Care.

[3] Carroll RW, Hall RM, Parry-Strong A, Wilson JM, Krebs JD (2013). Therapeutic options in the management of obesity. N Z Med J.

[4] Konrad FM, Kramer KM, Schroeder TH, Stubbig K (2011). [Anesthesia and bariatric surgery]. Anaesthesist.

[5] Nishiyama T, Kohno Y, Koishi K (2012). Anesthesia for bariatric surgery. Obes Surg.

[6] Ogunnaike BO, Jones SB, Jones DB, Provost D, Whitten CW (2002). Anesthetic considerations for bariatric surgery. Anesth Analg.

[7] Schumann R (2011). Anaesthesia for bariatric surgery. Best Pract Res Clin Anaesthesiol.

[8] Szczyrba M, Kaltofen H, Mols G (2009). [Anaesthesiological challenges in patients for bariatric surgery]. Zentralbl Chir.

[9] Bergland A, Gislason H, Raeder J (2008). Fast-track surgery for bariatric laparoscopic gastric bypass with focus on anaesthesia and peri-operative care. Experience with 500 cases. Acta Anaesthesiol Scand.

[10] Heinrich S, Horbach T, Salleck D, Birkholz T, Irouschek A, Schmidt J (2011). [Perioperative anaesthesiological management in 167 patients undergoing bariatric surgery]. Zentralbl Chir.

[11] Jacobsen HJ, Bergland A, Raeder J, Gislason HG (2012). High-volume bariatric surgery in a single center: safety, quality, cost-efficacy and teaching aspects in 2,000 consecutive cases. Obes Surg.

[12] Leykin Y, Pellis T, Del Mestro E, Fanti G, Marzano B (2008). Perioperative management of 195 consecutive bariatric patients. Eur J Anaesthesiol.

[13] Pierre S, Corno G, Benais H, Apfel CC (2004). A risk score-dependent antiemetic approach effectively reduces postoperative nausea and vomiting--a continuous quality improvement initiative. Can J Anaesth.

[14] Greenstein AJ, Wahed AS, Adeniji A, Courcoulas AP, Dakin G, Flum DR, Harrison V, Mitchell JE, O’Rourke R, Pomp A, Pender J, Ramanathan R, Wolfe BM (2012). Prevalence of adverse intraoperative events during obesity surgery and their sequelae. J Am Coll Surg.

[15] Shang E, Beck G (2009). [Special anaesthesiological requirements in bariatric surgery]. Anasthesiol Intensivmed Notfallmed Schmerzther.

[16] Huschak G, Kaisers UX (2011). [Anesthesia for bariatric surgery. Comorbidity determines the quality of results]. Anaesthesist.

[17] Ramaswamy A, Gonzalez R, Smith CD (2004). Extensive preoperative testing is not necessary in morbidly obese patients undergoing gastric bypass. J Gastrointest Surg.

[18] Neligan PJ, Porter S, Max B, Malhotra G, Greenblatt EP, Ochroch EA (2009). Obstructive sleep apnea is not a risk factor for difficult intubation in morbidly obese patients. Anesth Analg.

[19] Birkmeyer JD, Finks JF, O'Reilly A, Oerline M, Carlin AM, Nunn AR, Dimick J, Banerjee M, Birkmeyer NJ, Michigan Bariatric Surgery C (2013). Surgical skill and complication rates after bariatric surgery. N Engl J Med.

[20] Brodsky JB, Lemmens HJ, Brock-Utne JG, Vierra M, Saidman LJ (2002). Morbid obesity and tracheal intubation. Anesth Analg.

[21] Whalen FX, Gajic O, Thompson GB, Kendrick ML, Que FL, Williams BA, Joyner MJ, Hubmayr RD, Warner DO, Sprung J (2006). The effects of the alveolar recruitment maneuver and positive end-expiratory pressure on arterial oxygenation during laparoscopic bariatric surgery. Anesth Analg.

[22] Naguib M (2007). Sugammadex: another milestone in clinical neuromuscular pharmacology. Anesth Analg.

[23] Kramer CK, Zinman B, Retnakaran R (2013). Are metabolically healthy overweight and obesity benign conditions? A systematic review and meta-analysis. Ann Intern Med.

[24] Tung A (2010). Anaesthetic considerations with the metabolic syndrome. Br J Anaesth.

[CR25] The pre-publication history for this paper can be accessed here:http://www.biomedcentral.com/1471-2253/14/125/prepub

